# Identifying Leafhopper Targets for Controlling Aster Yellows in Carrots and Celery

**DOI:** 10.3390/insects11070411

**Published:** 2020-07-02

**Authors:** Patrick T. Stillson, Zsofia Szendrei

**Affiliations:** Department of Entomology, Michigan State University, East Lansing, MI 48823, USA; stillson@msu.edu

**Keywords:** Cicadellidae, insect vector, pest management, plant disease, phytoplasma

## Abstract

Aster yellows phytoplasma (*Candidatus Phytoplasma asteris*) is a multi-host plant pathogen and is transmitted by at least 24 leafhopper species. Pathogen management is complex and requires a thorough understanding of vector dynamics. In the American Midwest, aster yellows is of great concern for vegetable farmers who focus on controlling one vector, *Macrosteles quadrilineatus*—the aster leafhopper. However, vegetable-associated leafhopper communities can be diverse. To investigate whether additional species are important aster yellows vectors, we surveyed leafhopper communities at commercial celery and carrot farms in Michigan from 2018 to 2019 and conducted real-time PCR to determine infection status. Leafhoppers were collected within crop fields and field edges and identified with DNA barcoding. Overall, we collected 5049 leafhoppers, with the most abundant species being *M. quadrilineatus* (57%) and *Empoasca fabae*—the potato leafhopper (23%). Our results revealed the most abundant aster yellows vector in Michigan in both crops is *M. quadrilineatus,* but we also found that *E. fabae* may be a potential vector for this pathogen. While several taxa reside in and near these crops, we did not find strong evidence that they contribute to phytoplasma infection. These findings indicate that *M. quadrilineatus* should be the primary target for controlling this pathogen.

## 1. Introduction

Aster yellows phytoplasma (*Candidatus* Phytoplasma asteris) is an insect-vectored plant pathogen [1] which causes a variety of symptoms including yellowing, virescence, phyllody, witch’s broom, and ultimately premature death [2]. Even when infected crops reach harvest, they are often unmarketable [1,3]. Aster yellows has been reported to reduce yields by 10% [4] and is one of the most widespread phytoplasmas, affecting 14 vegetable crops across various plant families [5,6,7]. It is vectored by at least 24 leafhoppers [8], which must acquire the phytoplasma from the environment by feeding on infected plants [1], since phytoplasmas are rarely transovarial [1,9]. Not all leafhoppers can transmit aster yellows, which may be associated with a narrow diet breadth, where the leafhoppers do not feed on the infected plant or do not feed on the phloem of the infected plant [7]. If a leafhopper feeds on an infected plant, the phytoplasma must successfully migrate to the salivary glands before transmission is possible [10,11]. Polyphagous leafhoppers can acquire phytoplasma from crops or weedy host plants and then spread it among susceptible crop fields or between the field and field edge [7].

Movement of phytoplasmas in agroecosystems is primarily facilitated by polyphagous leafhoppers feeding locally on infected host plants [1], and seasonal migrations of some leafhopper species from overwintering to summer habitats [12,13,14]. In North America, the main vector of aster yellows phytoplasma is the migratory *Macrosteles quadrilineatus* (Forbes)—the aster leafhopper (Hemiptera: Cicadellidae), a polyphagous species with over 300 host plants [15], and a broad geographic distribution [12] *Macrosteles quadrilineatus* may move between different crops, between fields, and into field edges to feed on grasses and weeds [16]. This movement among various host plants can increase the chances of other leafhopper vectors acquiring aster yellows [1,17]. Currently, *M. quadrilineatus* is the focus for controlling aster yellows phytoplasma for vegetable farmers in the Midwest, USA. However, agroecosystems can have diverse leafhopper communities. These leafhopper vectors may then create disease reservoirs in the field edge, especially in perennial weeds that can be a source of infection every year [1,18,19]. The identity and vector status of these other leafhopper taxa is understudied and may be important for developing sustainable management methods for aster yellows.

To investigate whether additional leafhopper species are important aster yellows vectors, we collected leafhoppers from commercial celery and carrot farms in Michigan during the 2018 and 2019 growing seasons. We collected leafhoppers from the crops and the field edges using sweep nets, identified the leafhoppers to the lowest taxonomic level possible using morphological identifications and DNA barcoding, conducted molecular diagnostics to determine whether they contained phytoplasma, and compared leafhopper species abundances in the different crops and locations. In order to identify leafhoppers and to detect aster yellows phytoplasma, we used established molecular tools. We identified the collected leafhoppers using, morphological features, existing information on their geographic distribution and DNA barcoding due to its widespread use, ease, and accuracy [20]. We relied on the growing databases of DNA sequences from identified species [21] that improve insect identification, especially in the case of taxa where taxonomic experts are lacking. Additionally, real-time PCR was used to detect phytoplasma infected leafhoppers. In recent years, real-time PCR has become an established and powerful tool for detecting phytoplasmas, as they cannot be grown in pure culture [22]. Real-time PCR has high sensitivity and is able to detect phytoplasma concentrations down to a single cell. It is performed in a single reaction vessel, making this an efficient and useful tool in phytoplasmas detection [22,23].

## 2. Materials and Methods

### 2.1. Study System

**Figure 1:**
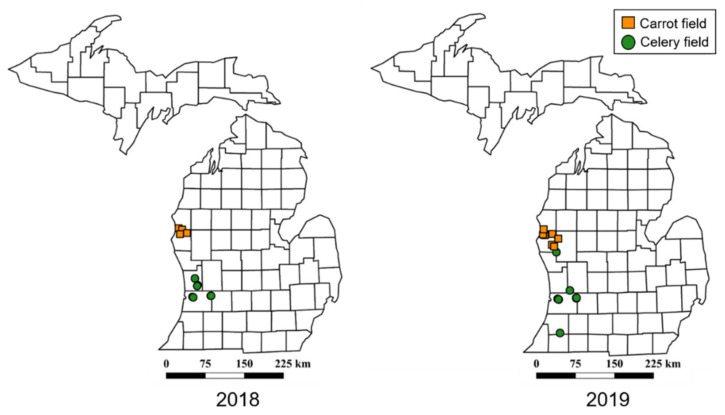
Map of collections sites from Michigan, USA. Symbols indicate locations of celery and carrot fields where leafhoppers were collected in 2018 and 2019. Leafhoppers were collected using sweep nets and transported to the laboratory for identification and to determine phytoplasma infectivity.

Leafhoppers were collected using sweep nets (38 cm diameter aerial net) from mid-May through early August in the 2018 and 2019 growing seasons. All farms surveyed (Figure 1) were large-scale commercial operations, managed with synthetic pesticides. Sweep net samples were taken between 11:00 and 14:00 on clear days when insecticides had not been recently applied.

**Figure 2:**
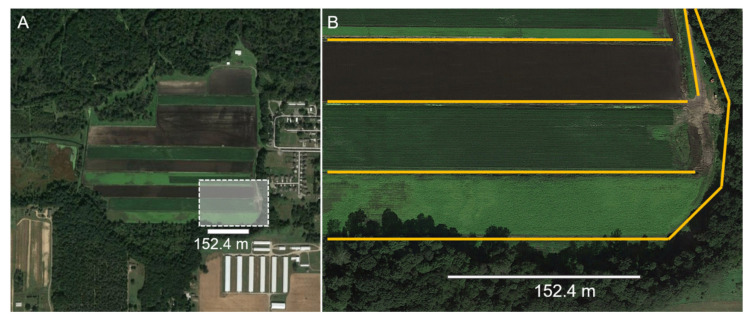
Leafhoppers were collected in celery and carrot field edges in Michigan in 2018 and 2019. (**A**) Aerial view of a celery field with boxed area magnified in B. (**B**) The surveyed field edge types are indicated by the yellow lines, consisting of vegetation between adjacent crop fields and edges between fields and non-agricultural vegetation, including weedy herbaceous plants growing along roads or paths adjacent to fields, or plants naturally growing along wooded edges.

In 2018, leafhoppers were collected three times from the field edge (26 June, 10 July, and 1 August) from one celery farm and weekly from inside seven celery and five carrot fields (*n* = 36 collections). In 2019, collections from both within the fields and from the edges were conducted weekly at ten celery and seven carrot farms (*n =* 226 collections). A minimum of 100 sweeps from inside the crop fields were taken from randomly chosen sites, approximately >10 m into the field, away from the field edge. The ‘field edge’ consisted of naturally occurring vegetation around crop fields, along driveways, or along wooded edges (Figure 2A,B). In both years, sweeps were taken within randomly selected 5 m sections of the field edge; the total number of sweeps varied by field edge due to the variability in the amount of vegetation available for sweeping (200–500 sweeps/field). After collection, all leafhoppers were transported in a cooler from the field to the laboratory, where they were stored at −20 °C.

### 2.2. Leafhopper Identification

In the laboratory, leafhoppers were sorted into several groups upon arrival from the field: *M. quadrilineatus*, *Empoasca fabae* (Harris)—the potato leafhopper, and other leafhoppers which were grouped based on morphological similarities. *Macrosteles quadrilineatus* and *E. fabae* were sight identified and were placed into homogenization tubes for DNA extraction. All *M. quadrilineatus* (*n =* 2883) DNA was extracted following Demeuse et al. [22]; modifications to this protocol included individually extracting DNA from each leafhopper and eluting DNA in 50 µL elution buffer (Qiagen, Valencia, CA, USA).

For all of the non-*M. quadrilineatus* leafhoppers, we used a modified Dellaporta DNA extraction [24]. This protocol does not utilize a spin column and thus DNA is preserved in better condition for sequencing compared to the protocol described in Demeuse et al. [22] that uses a spin column potentially leading to DNA fragmentation. Leafhoppers (*n =* 2166) were placed individually in 2 mL homogenization tubes (Sarstedt, Nümbrecht, Germany), along with 3 homogenization beads (2.3 mm diameter, zirconia/silica; BioSpec Products, Bartlesville, OK, USA), and 400 µL Dellaporta buffer (1 mL of 100 mM Tris, pH 8.0, 1 mL of 500 mM EDTA, 1.25 mL 500 mM NaCl, 10 µL β-mercaptoethanol and 6.75 mL of ultrapure water). Leafhoppers were homogenized for 10 s at 4.0 m/s (FastPrep-24, MP Biomedicals, Irvine, CA, USA). Afterwards, 52.8 µL 10% SDS was added, samples were vortexed then incubated at 65 °C for 10 min. After incubation, 128 µL 5 M potassium acetate was added. Samples were vortexed then centrifuged for 10 min at 15,000 rcf. Supernatant was removed and placed in a clean 1.7 mL centrifuge tube. Afterwards, 240 µL cold isopropanol was added to the supernatant and the samples were incubated at room temperature for 5 min. Samples were mixed by gentle inversion. Samples were placed in a −20 °C freezer for 1 h and then centrifuged in a 4 °C refrigerated centrifuge (Centrifuge 5810 R, Eppendorf, Hamburg, Germany) for 20 min at 15,200 rcf. Supernatant was removed and 800 µL 70% ethanol was added to the pelleted DNA. Samples were again mixed by gentle inversion and then placed back in the refrigerated centrifuge for 10 min at 15,200 rcf. The supernatant was removed, and pellets allowed to air dry. Pellets were suspended in 50 µL elution buffer (Qiagen).

We used conventional PCR to amplify the cytochrome c oxidase subunit I (COI) gene using the Ron and Nancy primer set (Thermo Fisher Scientific, Waltham, MA, USA) [25] from 1 to 15 leafhoppers per morphotype based on the availability of leafhoppers per morphotype. We then cleaned the PCR product with QIAquick PCR Purification Kit (Qiagen) and submitted the DNA to Michigan State University’s Research Technology Support Facility (RTSF) for Sanger sequencing. The sequences were compared to the National Center for Biotechnology Information genomic database (NCBI), and the leafhoppers were identified based on sequence match. The top result was accepted as a positive identification when the query sequence had a 95–100% sequence match, if it morphologically matched the collected leafhopper, and if the species’ known geographic distribution overlapped with Michigan. In cases where the top result was rejected, we searched through the list of >80% sequence matches for taxa that were acceptable based on morphology and geographic distribution. We identified morphotypes to the genus level (i.e., *Agallia* sp.), if the subset of sequenced leafhoppers from the same morphotype group were identified as different species, suggesting that multiple species make up that morphotype. For identifying leafhoppers based on morphology, we used previously identified leafhoppers from the Albert J. Cook Arthropod Research Collection at Michigan State University and Bug Guide [26]. For three morphotype groups (*Athysanus argentarius* (Metcalf), *Idiocerus raphus* (Freytag), and *Norvellina* sp.), only a few leafhoppers were collected, and these consistently provided poor sequencing results. These leafhoppers were only identified based on morphological comparisons to leafhoppers found in the Albert J. Cook Arthropod Research Collection. Morphological features used for comparisons included body and wing coloration, markings on the head, size, and wing venation. Some morphotype groups could not be identified, and thus they were recorded as unidentified.

For those leafhoppers that were identified to the species level, individuals were counted and recorded by species. Unidentified morphotypes were grouped as unknown Cicadellidae and their numbers recorded. Leafhoppers were divided into commonly collected (≥50 leafhoppers collected) or rare (<50 leafhoppers collected) taxa. For future reference, one adult specimen of each morphological group was pinned, or one nymph was preserved in 70% ethanol. Voucher specimens for all identified leafhoppers were stored in the Albert J. Cook Arthropod Research Collection, Michigan State University (voucher number: 2019-09), except for *Erythroneura* sp., morphotype 15, and 16 (Figure S1–S3). Sequences used for identification were deposited on NCBI GenBank with accession numbers MT643826-MT643893 (Table S1).

### 2.3. Detection of Phytoplasma

All leafhoppers were evaluated for the presence of aster yellows phytoplasma with a real-time PCR TaqMan assay [22], using universal phytoplasma primers and probe [23] (Thermo Fisher Scientific). Leafhoppers were tested in duplicate, and if one of the replicates tested positive for the presence of phytoplasma (cycle threshold (*C*_t_) value ≤40) and the other was negative, the leafhopper was retested. For *M. quadrilineatus*, we used a *C*_t_ value <32 to determine positives, as established for our regular diagnostic work for farmers [22]. All non-*M. quadrilineatus* with *C*_t_ values ≤40, were also tested with conventional PCR using P3/P7 universal phytoplasma primers [27] to verify the presence of phytoplasma. The PCR products were run on a 1% agarose gel precast with GelRed (Biotium, Fremont, CA, USA) for 1 h at 90 V. Bands were visualized with a UV transilluminator (Bioolympics, Thousand Oaks, CA, USA). In addition, we searched the literature to determine which of the collected leafhoppers are known vectors for aster yellows phytoplasma or other phytoplasmas, or if there are congeners that are phytoplasma vectors. Vector status for leafhoppers found through the literature search was determined through transmission studies where leafhoppers inoculated healthy test plants or inoculated sucrose solutions. We then compared the collected leafhopper species to this list of documented vectors.

### 2.4. Data Analysis

To identify which leafhopper species may be feeding on the crops, or moving between the crops and field edge, we determined whether there were differences in leafhopper species abundance between species found in both locations and crops. We used a generalized linear model, where crop type and field location were fixed factors. Differences among means of tested factors were determined with post-hoc pairwise comparison (Tukey’s HSD: α = 0.05; function = ‘emmeans’, package = ‘emmeans’) [28]. The total number of leafhoppers per 100 sweeps was used for each of the most abundant leafhopper species (genera or species ≥50 leafhoppers collected). Leafhoppers per 100 sweeps was used to standardize leafhopper densities across collections with different numbers of sweeps. We performed separate statistical analyses for celery and carrot.

To determine whether there were differences in the number of infected *M. quadrilineatus* between the crop and field edge, we used a generalized linear model, where field location (inside or outside field) was used as a fixed factor. Differences among means of tested factors was again determined with post-hoc pairwise comparison (Tukey’s HSD: α = 0.05). In addition, this was also performed using crop (carrot or celery) as a fixed factor. All statistical analyses were conducted in R v.3.6.0 [29]. Data used in statistical analyses is provided in Table S2.

### 2.5. Phylogenetic Analysis

Gene sequences (COI) were aligned using MAFFT version 7.450 [30] with the default parameters. Maximum-likelihood phylogenetic trees were generated with RAxML version 8.2.10 [31] using a GTR+Gamma model on the CIPRES Science Gateway [32]. Node support values were calculated using rapid bootstrapping with 500 replicates.

## 3. Results

**Figure 3:**
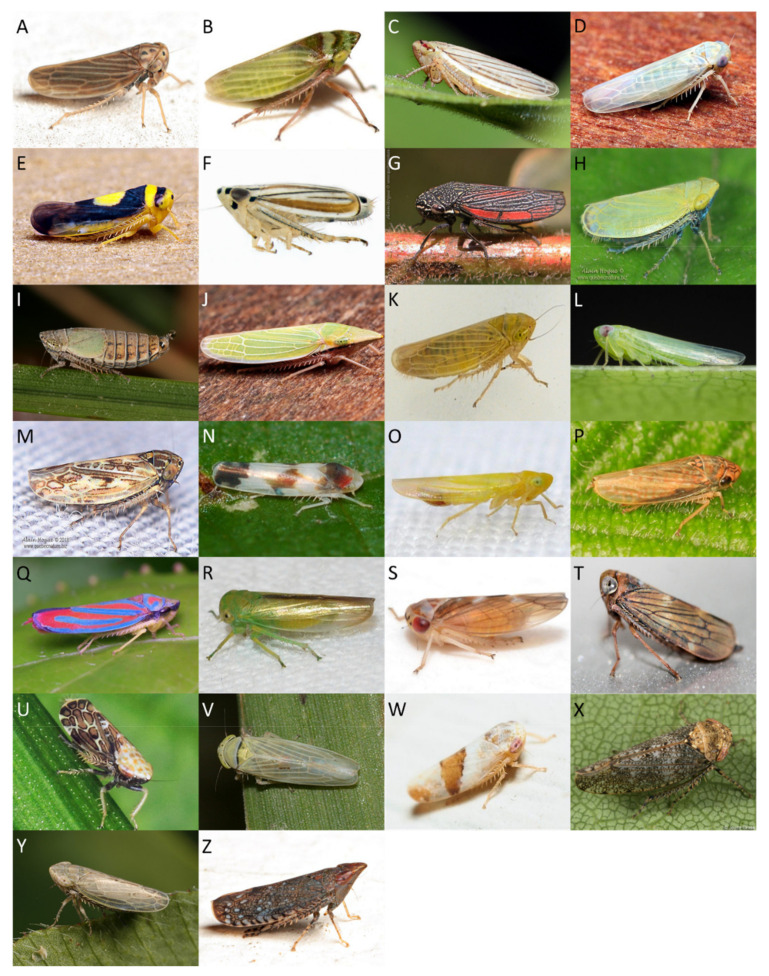
Leafhoppers collected from Michigan, USA, celery and carrot farms in the period 2018–2019. (**A**) *Agallia* sp., (**B**) *Aphrodes bicinctus*, (**C**) *Athysanus argentarius*, (**D**) *Balclutha* sp., (**E**) *Colladonus clitellarius*, (**F**) *Commellus* sp., (**G**) *Cuerna* sp. *, (**H**) *Diplocolenus* subg. *verdanus*, (**I**) *Doratura stylata*, (**J**) *Draeculacephala* sp., (**K**) *Elymana inornata*, (**L**) *Empoasca fabae*, (**M**) *Endria inimica*, (**N**) *Erythroneura* sp., (**O**) *Forcipata loca*, (**P**) *Graphocephala hieroglyphica* *, (**Q**) *Graphocephala* sp., (**R**) *Idiocerus raphus*, (**S**) *Idiocerus* sp., (**T**) *Jikradia olitoria*, (**U**) *Latalus* sp., (**V**) *Macrosteles quadrilineatus*, (**W**) *Norvellina* sp., (**X**) *Paraphlepsius* sp., (**Y**) *Psammotettix lividellus*, and (**Z**) *Scaphytopius* sp. Note: * indicates that only nymphs were collected; all other leafhoppers were collected as adults or as both adults and nymphs. See acknowledgements for photo credits.

In total, we collected 5049 leafhoppers from celery and carrot fields and their field edges combined during the 2018 and 2019 growing seasons. We identified 25 leafhopper taxa with 14 identified to the species level, 11 identified to the genus level, and 16 morphotypes identified to family level (Cicadellidae; Figure 3A–Z, Table 1). Out of the common taxa that represented 94% of collected leafhoppers, eight were identified (five were identified to the species level and three to the genus level; Table 1). The most abundant species were *M. quadrilineatus* (57%) and *E. fabae* (23%).

### 3.1. Carrot Leafhopper Collections

**Figure 4:**
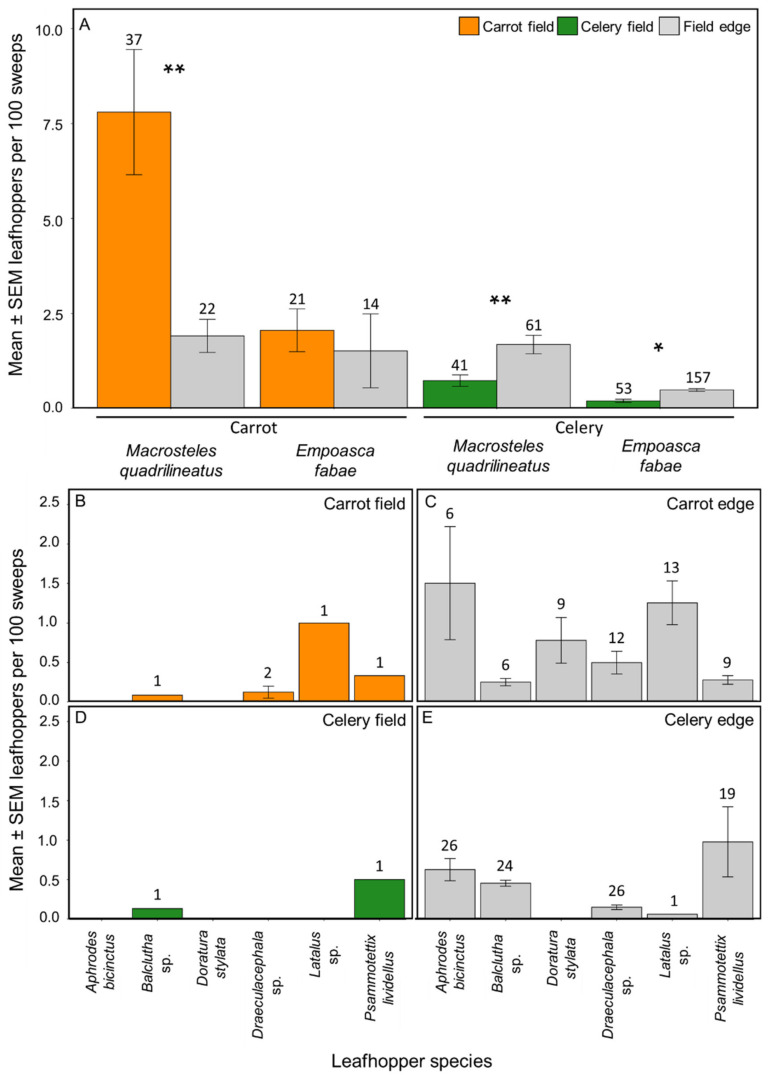
Mean ± SEM leafhoppers per 100 sweeps of the eight most abundant leafhopper taxa (≥50 individuals collected) from commercial carrot and celery fields and field edges in Michigan in 2018 and 2019. *Macrosteles quadrilineatus* and *Empoasca fabae* were the most commonly collected species (**A**). Six other abundant leafhopper taxa (≥50 individuals collected of each) were represented in our samples from carrot fields (**B**), carrot field edges (**C**), celery fields (**D**), and celery field edges (**E**). Numbers above bars indicate the number of collections for each taxa and location. Asterisks indicate statistically significant differences between field and edge collections for each taxa (Tukey’s HSD; * *p*-value ≤ 0.05, ** *p*-value ≤ 0.01).

We collected 2995 leafhoppers from carrot farms in 2018 and 2019, with 1932 leafhoppers (65%) collected from within carrot fields and 1063 (35%) from the field edges. A total of 23 taxa were identified with 13 identified to the species level and 10 identified to the genus level. The most abundant leafhopper taxa were *M. quadrilineatus* (62%), *E. fabae* (16%), *Doratura* *stylata* (Boheman) (6%), and *Latalus* sp. (5%) (≥50 individuals collected for each). Leafhoppers found only in carrots included *Commellus* sp., *Cuerna* sp., *Diplocolenus* subg. *verdanus*, *Doratura stylata*, *Elymana inornata* (Van Duzee), *Endria inimica* (Say), and *Graphocephala hieroglyphica* (Say). When comparing the abundances of the eight most abundant leafhopper taxa within and around carrot fields, *M. quadrilineatus* had 1.75-fold greater abundance within the carrot fields than in the field edge (*p*-value ≤ 0.01), *E. fabae* had 4.20-fold greater abundance within the field (*p*-value = 0.99; Figure 4A), as did *Psammotettix* *lividellus* (Zetterstedt) with 1.44-fold greater abundance in the field (*p*-value = 0.99) than in the field edge. Conversely, *Latalus* sp., *Balclutha* sp., and *Draeculacephala* sp. had greater abundances within the field edges than in the carrot fields, with 3.00- (*p*-value = 0.97), 2.00- (*p*-value = 0.99), and 1.94- (*p*-value = 0.78) fold more leafhoppers collected respectively. Two other species—*Aphrodes bicinctus* (Schrank) and *D. stylata*—were only found in the carrot field edge (Figure 4B,C).

### 3.2. Celery Leafhopper Collections

We collected 2054 leafhoppers from 2018 and 2019 from celery farms, with 701 leafhoppers (34% of the total) collected from within the celery fields and 1353 (66%) from the field edge. A total of 18 taxa were identified, with 9 identified to the species level and 9 identified to the genus level. *Macrosteles* *quadrilineatus* (50%), *E. fabae* (32%), and *P. lividellus* (9%) were the most abundant leafhopper taxa (≥50 individuals collected of each). *Erythroneura* sp. was only found in celery fields but not in field edges. When comparing the abundances of the eight most abundant leafhopper taxa within and outside celery fields, they were all predominantly found in the field edge. *Macrosteles quadrilineatus* was 1.65-fold more abundant in celery field edges than within the field (*p*-value ≤ 0.01), similarly, *E. fabae* was 2.23-fold more abundant in field edges than within celery fields (*p*-value = 0.03; Figure 4A). *Psammotettix lividellus* and *Balclutha* sp. were both found primarily outside celery fields with 1.13- (*p*-value = 0.97) and 1.21- (*p*-value = 0.97) fold greater abundances in the edge respectively than in the celery field. Three other taxa—*Latalus* sp., *A. bicinctus*, and *Draeculacephala* sp.—were found only in the field edge (Figure 4D,E). *Doratura stylata* was absent from celery fields and edge collections.

### 3.3. Phytoplasma Infectivity

Across the two study years, 12 *M. quadrilineatus* tested positive for aster yellows using the *C*_t_ value threshold of 32 typically used in our detection assay [22]. Twenty-seven individuals from nine other taxa had *C*_t_ values between 25.2 and 40: 16 *E. fabae*, 4 *Latalus* sp., 1 *Balclutha* sp., 1 *Draeculacephala* sp., 1 *D. stylata*, 1 *Graphocephala* sp., 1 *Idiocerus* sp., 1 *P. lividellus*, and 1 *Scaphytopius* sp. (Table 1). One *E. fabae* tested positive for aster yellows phytoplasma using P3/P7 primers, while all the other leafhoppers were negative for aster yellows with this primer set.

In addition, we found three known aster yellows phytoplasma vectors in our collections including *A. bicinctus*, *Athysanus argentarius*, and *E. inimica* but none of these produced *C*_t_ values≤ 40. Of the leafhoppers that were identified to genus, *Agallia* sp., *Paraphlepsius* sp., and *Scaphytopius* sp. may potentially be vectors since there are aster yellows vectors in these genera. Of those we identified to the species level, *Colladonus clitellarius* (Say), *E. inornata*, and *G. hieroglyphica* while not known to transmit aster yellows, other species in their genera are aster yellows vectors ( Table 2; Table 3).

There was no difference in the number of infected *M. quadrilineatus* between crops (*p*-value = 0.61) or between the field and the field edge (*p*-value = 0.67).

### 3.4. Phylogenetic Analysis

According to the generated maximum-likelihood phylogeny (Figure S4), the COI-identified leafhoppers were placed with the same taxa, except for five sequences found on the top of the tree (1 *Cuerna* sp., 2 *E. fabae*, and 2 *Paraphlepsius* spp.). These sequences did not fall into the appropriate groups possibly due to low quality sequencing. Additionally, from this analysis, we generated a sequence dissimilarity matrix listing the number of nucleotide differences and overall percent difference between all COI sequences (Table S3). When leafhopper sequences were observed within the same clade, there was less than 10% difference between the sequences, while they had 10–30% differences between neighboring clades or branches. This provides evidence that our COI barcoding was effectively able to distinguish different leafhopper species.

## 4. Discussion

Our leafhopper survey confirmed that *M. quadrilineatus* is the primary leafhopper vector of aster yellows phytoplasma in Michigan celery and carrot agroecosystems, which is consistent with findings from Ohio [12] and Wisconsin [78] carrot fields, and is the first study to confirm this in Midwestern celery fields. While other leafhopper species reside in and near these crops, we did not find strong evidence that they contribute to phytoplasma infections within these crops. Additionally, we determined that the leafhopper communities were different between the two agroecosystems with the field edges characterized by a greater diversity of species than the crop fields.

With aster yellows phytoplasma’s wide host plant range [5], it is essential to identify its leafhopper vectors that may threaten crops. Our results indicated that across both celery and carrot cropping systems, *M. quadrilineatus* was the most abundant species*,* and although carrots overall had more diversity in leafhopper taxa, the edges of both crops were comparable in leafhopper abundance and composition. The known aster yellows phytoplasma vectors collected were *A. bicinctus*, *A. argentarius*, and *E. inimica* (only in carrot) which were all found in the field edge and are known to feed on grasses, cereals, and clover [35,42,79]. We also collected *Scaphytopius* sp. from both cropping systems and while they are likely to be *Scaphytopius acutus* (Say), a known vector of aster yellows [80], we did not find strong evidence that this leafhopper is vectoring phytoplasma (*C*_t_ = 39.3, *n =* 1). Unlike some of the other leafhopper species in our collections, *Scaphytopius* sp. was found in both carrot and celery fields and field edges, indicating that it is likely to frequently move to new host plants. *Doratura stylata* and *Latalus* sp. had the lowest *C*_t_ values (*C*_t_ = 34.93, *n =* 1; *C*_t_ = 35.39–37.03, *n =* 4), besides *M. quadrilineatus* and *E. fabae*. While *D. stylata* has not been reported in the literature as a phytoplasma vector, *Latalus* sp. has been reported as a vector, but we were unable to verify the real-time PCR findings with conventional PCR and sequencing. High *C*_t_ values can potentially result when non-vector leafhoppers feed on an infected plant and the phytoplasma is present in the digestive tract [81]. Because of this, the only way to confirm vector status is through transmission assays which involve feeding suspected vectors on phytoplasma infected plants to acquire the pathogen, and having them inoculate healthy plants or a sucrose solution [48,80,82]. If the disease is detected in the plant, after a latency period lasting up to a month, or in the sucrose solution after inoculation, then the leafhopper is a vector for the phytoplasma [48,80,82]. Nevertheless, real-time PCR is known to be more sensitive for aster yellows detection than conventional PCR [22]; thus, we cannot exclude the possibility that some of the leafhoppers with high *C*_t_ values are in the early stages of infection.

We verified one *E. fabae* with conventional PCR and sequencing as containing aster yellows phytoplasma. *Empoasca fabae* has previously been detected with a strain of aster yellows, although the authors did not confirm vector status using bioassays [83]. Two other *Empoasca* spp. are known phytoplasma vectors: *Empoasca papayae* (Oman) vectors Papaya Bunchy Top associated with *Candidatus* Phytoplasma aurantifolia [45,47,84]*,* and *Empoasca decipiens* (Paoli) vectors chrysanthemum yellows phytoplasma, which is closely related to aster yellows (Table 2) [46].

Although we hypothesized that field edges may be disease reservoirs and a source of infection for the crops, our findings indicate that the edge may not be the primary source of phytoplasma infection. Since more *M. quadrilineatus* were positive for aster yellows in samples collected from within crop fields, compared to field edges, this could indicate that aster yellows phytoplasma is brought into the field by migrating *M. quadrilineatus* that later move from the crop to the field edge [33,34]. In the field edge, infected *M. quadrilineatus* can infect plants which may become disease reservoirs and sources of aster yellows phytoplasma for other leafhoppers.

Based on our findings, *M. quadrilineatus* had higher abundance in carrot fields compared to celery which could be due to differences in the management of the two crops. For example, celery is transplanted in the spring, while carrots are direct seeded, and thus celery is available earlier in the season for *M. quadrilineatus* colonization than carrots. Carrots are grown in counties North of the celery producing area (Figure 1), accentuating the difference in developmental stages between the two crops. The management intensity of the two crops is also different with more frequent insecticide applications in celery compared to carrots (personal observation, Z. Szendrei). This is likely due to the direct damage to celery stems and leaves by annually occurring pests such as caterpillars and aphids and the relatively higher value of celery compared to carrots ($19.5 million and $14.5 million respectively in Michigan in 2018) [85].

Areas surrounding crop fields such as field edges can play an important role in the lifecycle of vectored pathogens not only for creating disease reservoirs but for managing vector populations using trap crops [86]. Differences in host plant preference could be used to attract *M. quadrilineatus* away from crops to trap crops planted in field edges. For trap crops to work effectively, we first need to identify plants that are more attractive to *M. quadrilineatus* compared to the vegetable crops [87]. By planting trap crops, we may be able to mostly contain the leafhoppers in the field edge, especially when crops are in their most susceptible developmental stages. It will also be important to screen for aster yellows-resistant hosts, from which phytoplasma cannot be acquired and transmitted to other plants. Since other leafhopper species are likely not as important in aster yellows transmission, focusing on *M. quadrilineatus* behavioral management could potentially be an effective and sustainable strategy to reduce aster yellows’ economic impact. In addition, farmers may implement other control measures, such as mowing weedy field margins, thus reducing potential alternative hosts for both aster yellows and *M. quadrilineatus*. These management strategies can also be paired with diagnostics based support tools that inform growers about leafhopper infectivity [88]. By utilizing multiple methods for management, farmers will be able to better control aster yellows phytoplasma in a sustainable way.

## 5. Conclusions

Insect-vectored plant pathogens are challenging to manage with sustainable methods, especially when both the vector and pathogen have wide host ranges. Here, we made an important first step by confirming that *M. quadrilineatus* is an important vector of aster yellows and that *E. fabae* may potentially be another vector in celery and carrot agroecosystems. The next step will be to conduct transmission tests to determine whether *E. fabae* can vector aster yellows, as they are often abundant in aster yellows-susceptible crops—many of which also have *M. quadrilineatus* [89,90]. Both leafhoppers are found in the fields and field edges of celery and carrot fields, and if *E. fabae* can vector aster yellows, limiting where the leafhoppers can acquire the pathogen by using disease-resistant trap crops will minimize phytoplasma prevalence.

## Figures and Tables

**Table 1:** Leafhoppers collected from commercial celery and carrot farms in Michigan, USA, from 2018 to 2019. Field edges were defined as areas bordering the crop field or between adjacent fields where crops were not growing. Fractions indicate the number of individuals that generated cycle thresholds (*C*_t_ ≤ 40) in a real-time PCR with universal phytoplasma primers [23] out of the total number of individuals collected.

Genera/Species	Celery Field	Celery Edge	Carrot Field	Carrot Edge	2018 Total	2019 Total	*C*_t_ Value or Range
*Agallia* sp.	2	7	10	24	5	38	-
Aphrodes bicinctus ^†^	0	49	0	29	9	69	-
*Athysanus argentarius*	0	2	0	6	0	8	-
*Balclutha* sp. ^†^	1	29	1/2	23	1/21	34	36.97
*Colladonus clitellarius*	1	0	0	2	1	2	-
*Commellus* sp.	0	0	0	2	0	2	-
*Cuerna* sp.	0	0	1	2	0	3	-
*Diplocolenus* subg. *verdanus*	0	0	0	32	0	32	-
*Doratura stylata* ^†^	0	0	0	1/191	0	1/191	34.93
*Draeculacephala* sp. ^†^	0	1/23	3	45	1	1/70	39.90
*Elymana inornata*	0	0	0	2	0	2	-
*Empoasca fabae* ^†^	6/235	6/418	409	75	11/304	5/833	25.20–40.00
*Endria inimica*	0	0	0	6	0	6	-
*Erythroneura* sp.	1	0	0	0	1	0	-
*Forcipata loca*	0	8	0	1	0	9	-
*Graphocephala hieroglyphica*	0	0	41	5	0	46	-
*Graphocephala* sp.	0	1/1	27	1	1/28	1	40.00
*Idiocerus raphus*	3	1	0	1	3	2	-
*Idiocerus* sp.	0	1/11	0	0	1/10	1	36.51
*Jikradia olitoria*	0	6	0	2	0	8	-
*Latalus* sp. ^†^	0	2	2	4/135	0	4/139	35.39–37.03
*Macrosteles quadrilineatus* ^†^	1/447	3/582	7/1423	1/431	3/707	9/2176	17.56–31.73
*Norvellina* sp.	1	1	0	0	1	1	-
*Paraphlepsius* sp.	0	11	5	2	2	16	-
*Psammotettix lividellus* ^†^	8	1/187	1	18	0	1/214	36.93
*Scaphytopius* sp.	2	10	1/2	4	1/4	14	39.30
Unknown Cicadellidae	0	5	6	24	4	31	-
Total leafhoppers collected	7/701	13/1353	9/1932	6/1063	18/1101	21/3948	

^†^ Indicates the eight most abundant leafhopper taxa (≥50 individuals collected of each).

**Table 2:** Known leafhopper vectors of aster yellows phytoplasma or other phytoplasmas for the leafhoppers collected in this study. Phytoplasma abbreviations are AWB = alfalfa witches broom, AshY = ash yellows, AYp = aster yellows, Cp = clover phyllody, CYE = clover yellow edge, EastX = Eastern X, EAYp = European aster yellows, GFD = Grape flavescence doree, NAGVY = North American grapevine yellows IIIB, Sp = stolbur, and SGP = strawberry green petal. *Diplocolenus* subg. *verdanus*, *Doratura stylata*, *Forcipata loca* (DeLong & Caldwell), and *Idiocerus raphus* were omitted as there is no record of whether they or their congeners vector phytoplasmas.

Species	Vectors AYp	Vectors Other Phytoplasmas	Congener Vectors AYp	Congener Vectors Other Phytoplasmas	References
*Aphrodes bicinctus*	Yes	Cp, CYE, EAYp, SGP, Sp	-	*A. albifrons*	[8,33,34]
*Athysanus argentarius*	Yes	-	-	-	[35]
*Colladonus clitellarius*	- ^†^	AshY, EastX	*C. geminatus,* *C. montanus montanus*	*C. geminatus,* *C. montanus montanus*	[8,36,37,38,39,40,41]
*Elymana inornata*	-	-	*E. sulphurella*	*E. virescens*	[42,43,44]
*Empoasca fabae*	-	-	-	*E. decipiens, E. papayae*	[45,46,47]
*Endria inimica*	Yes	-	-	-	[48]
*Graphocephala hieroglyphica*	-	AWB	*G. severini*	*G. confluens, G. severini*	[8,49,50]
*Jikradia olitoria*	-	NAGVY	-	-	[51]
*Macrosteles quadrilineatus*	Yes	Cp, EAYp, Sp	*M. sexnotatus*	*M. cirstata*, *M. laevis*, *M. quadripunctulatus*, *M. sexnotatus*, *M. striifrons*, *M. viridigriseus*	[19,33,52,53,54,55,56,57,58,59,60,61,62,63,64,65]
*Psammotettix lividellus*	-	GFD	-	*P. cephalotes*, *P. striatus*	[8,18,66]

^†^ Indicates that no data is available about whether the species or congeners can vector AYp or other phytoplasmas.

**Table 3:** Known leafhopper vectors of aster yellows phytoplasma or other phytoplasmas for the leafhoppers collected in this study. *Commellus* sp., *Draeculacephala* sp., and *Erythroneura* sp. were omitted as there is no record of whether species in these genera vector phytoplasmas.

Genus	Vectors AYp	Vectors Other Phytoplasmas	References
*Agallia* sp.	*A. constricta*	-	[67,68]
*Balclutha* sp.	- ^†^	*B. punctata*	[69]
*Cuerna* sp.	-	*C. septentrionalis*	[50]
*Latalus* sp.	-	*Latalus* sp.	[70]
*Norvellina* sp.	-	*N. seminuda*	[51]
*Paraphlepsius* sp.	*P. apertinus, P. irroratus*	*P. irroratus*	[8,36,68,71]
*Scaphytopius* sp.	*S. acutus acutus, S. acutus delongi*	*S. acutus acutus*, *S. acutus delongi*, *S. magdalensis*	[8,36,40,72,73,74,75,76,77]

^†^ Indicates that no data is available about whether the genera can vector AYp or other phytoplasmas.

## Supplementary Materials

The following are available online at https://www.mdpi.com/2075-4450/11/7/411/s1, Figure S1: *Erythroneura* sp. voucher specimen, Figure S2: Morphotype 15 voucher specimen, Figure S3: Morphotype 16 voucher specimen, Figure S4: Phylogenetic tree of leafhopper COI sequences, Table S1: Accession number data, Table S2: Data used in analyses, Table S3: Pairwise dissimilarity matrix.
