# Supplementary material for: Identifying Leafhopper Targets for Controlling Aster Yellows in Carrots and Celery
Source: Insects. 2020 Jul 2;11(7):411. doi: 10.3390/insects11070411 (PMC7412092; doi:10.3390/insects11070411)
Supplement: Supplementary file 1 [file insects-11-00411-s001.zip › Figure S1-S4.docx]

**
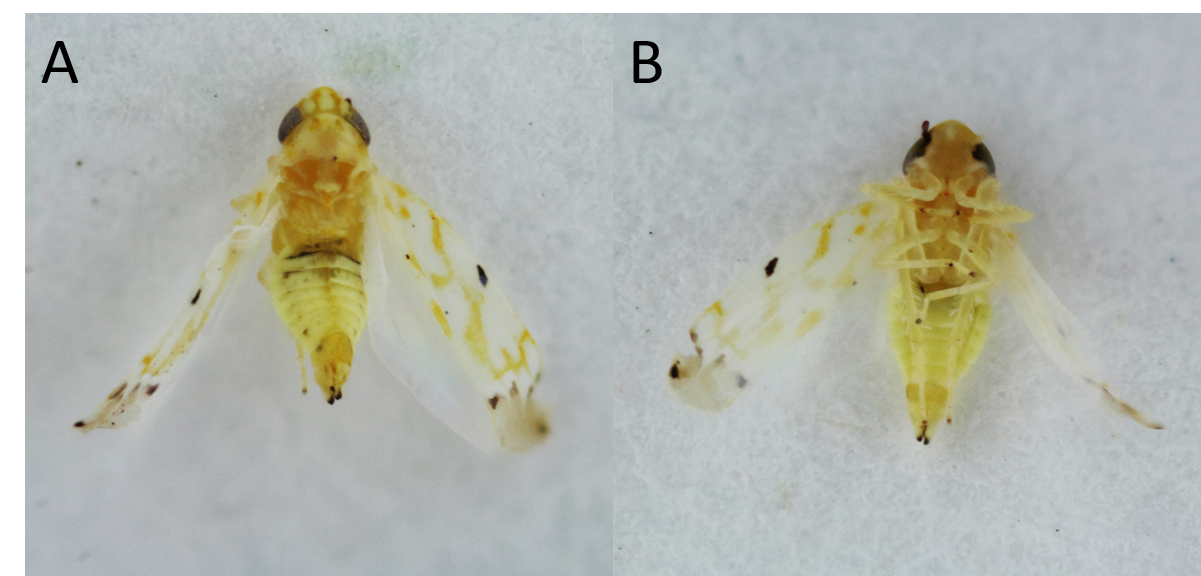
**

**Figure S1.** *Erythroneura* sp*.* voucher specimen. Dorsal (A) and ventral (B) view*.*


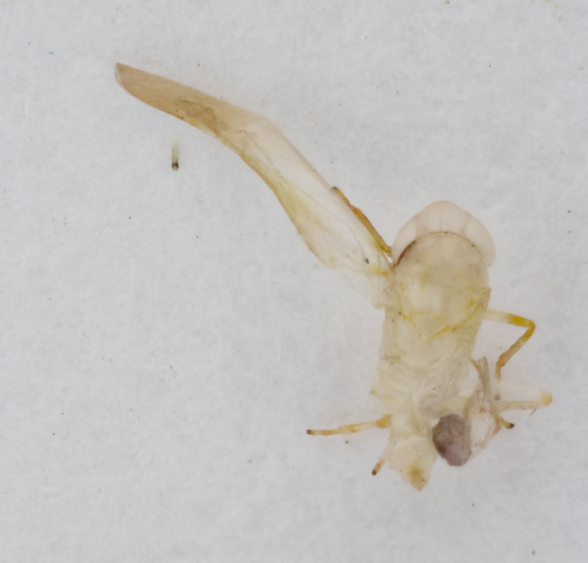


**Figure S2.** Morphotype 15 voucher specimen. Dorsal view.

**
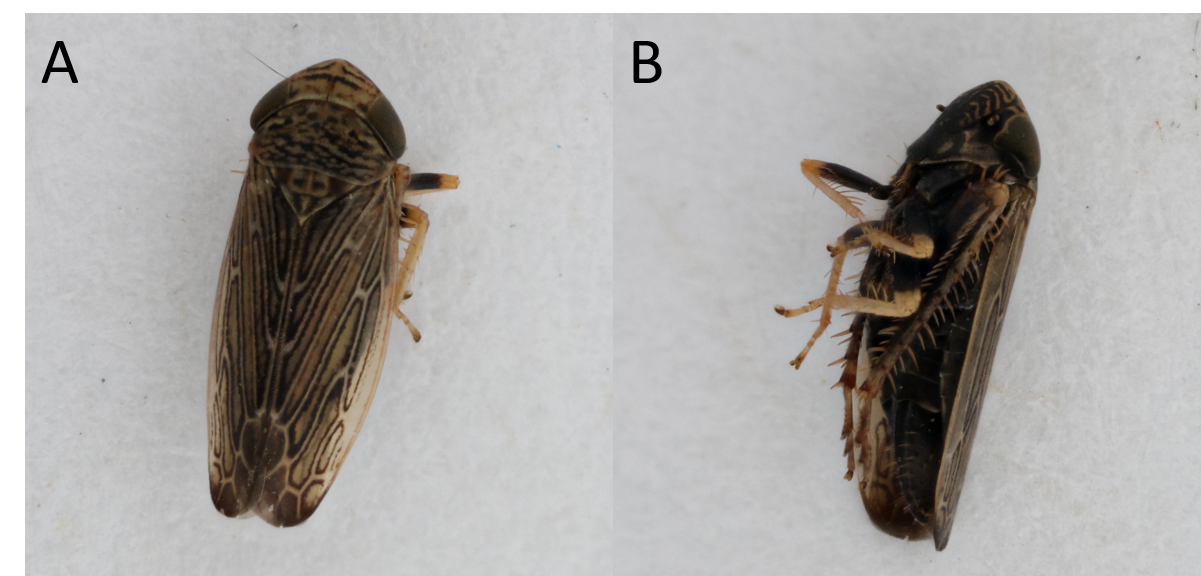
**

**Figure S3.** Morphotype 16 voucher specimen. Dorsal (A) and lateral (B) view.


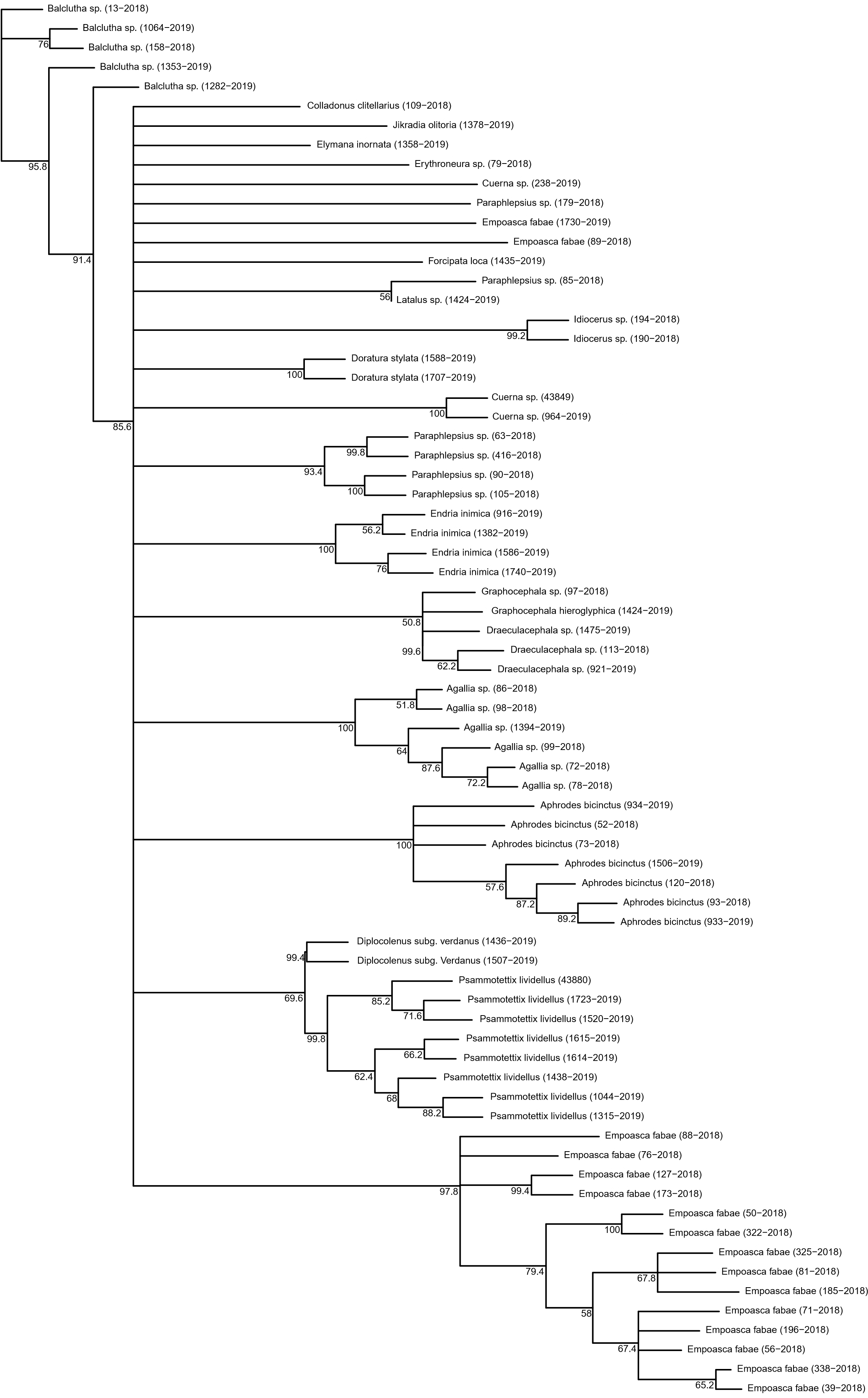


**Figure S4. Phylogenetic tree of leafhopper COI sequences**

Consensus maximum-likelihood phylogeny of the leafhopper COI sequences labeled with the taxa name and species voucher number in parentheses. Node support values were calculated using rapid bootstrapping which was halted automatically based on the MRE
