# Supplementary material for: Identifying Leafhopper Targets for Controlling Aster Yellows in Carrots and Celery
Source: Insects. 2020 Jul 2;11(7):411. doi: 10.3390/insects11070411 (PMC7412092; doi:10.3390/insects11070411)
Supplement: Supplementary file 1 [file insects-11-00411-s001.zip › Table S3 (pairwise disimilarity matrix).pdf]

Table S3. Pairwise sequence differences between COI regions sequenced from collected leafhopper species (upper right - number of nucleotide differences, lower left - percent difference between sequences).

		Seq1	Seq33	Seq52	Seq55	Seq53	Seq2	Seq30	Seq67	Seq50	Seq54	Seq71	Seq63	Seq70	Seq62	Seq66	Seq29	Seq10	Seq27	Seq59	Seq19	Seq26	Seq14	Seq17	Seq68	Seq58	Seq45	Seq56
Seq1	Balclutha sp. (13-2018)	-	0	2	4	75	96	94	93	93	93	78	93	78	90	87	92	87	87	87	87	87	87	95	95	95	96	101
Seq33	Balclutha sp. (158-2018)	0.00	-	0	2	78	104	96	95	93	93	76	93	76	116	87	94	88	88	88	88	88	88	94	94	94	95	103
Seq52	Balclutha sp. (1064-2019)	0.45	0.00	-	6	82	104	102	101	98	98	76	98	76	130	91	98	92	92	92	93	93	94	98	98	94	100	107
Seq55	Balclutha sp. (1353-2019)	0.91	0.42	1.22	-	77	106	101	100	97	97	77	96	77	133	90	96	91	91	91	91	91	93	95	95	99	98	104
Seq53	Balclutha sp. (1282-2019)	17.01	16.53	16.70	15.65	-	118	108	108	102	102	76	101	78	136	94	93	99	99	99	99	99	101	94	94	98	97	102
Seq2	Psammotettix lividellus (43880)	21.77	22.81	22.51	22.94	25.54	-	23	19	20	20	5	20	6	90	83	97	100	100	100	100	100	100	97	97	97	96	106
Seq30	Psammotettix lividellus (1723-2019)	23.27	21.67	22.37	22.10	23.63	5.39	-	6	9	9	8	12	9	114	73	86	90	90	90	92	92	90	90	90	93	88	91
Seq67	Psammotettix lividellus (1520-2019)	22.96	21.40	22.10	21.83	23.58	4.44	1.32	-	9	9	8	12	9	113	72	87	91	91	91	93	93	91	87	87	90	85	93
Seq50	Psammotettix lividellus (1044-2019)	21.09	19.70	19.96	19.72	20.73	4.33	1.98	1.97	-	0	0	3	1	114	71	84	87	87	87	89	89	87	85	85	88	84	91
Seq54	Psammotettix lividellus (1315-2019)	21.09	19.70	19.96	19.72	20.73	4.33	1.98	1.97	0.00	-	0	3	1	114	71	84	87	87	87	89	89	87	85	85	88	84	91
Seq71	Psammotettix lividellus (1615-2019)	22.22	22.16	21.78	22.06	21.78	1.43	2.55	2.54	0.00	0.00	-	0	1	59	58	65	71	71	71	71	71	71	66	66	66	67	72
Seq63	Psammotettix lividellus (1438-2019)	21.09	19.70	19.92	19.47	20.49	4.33	2.62	2.61	0.61	0.61	0.00	-	1	117	72	83	88	88	88	86	86	90	84	84	88	87	90
Seq70	Psammotettix lividellus (1614-2019)	22.03	21.97	21.59	21.87	22.16	1.70	2.84	2.83	0.28	0.28	0.29	0.28	-	60	59	67	72	72	72	72	72	72	68	68	68	69	73
Seq62	Diplocolenus subg. verdanus (1436-2019)	20.41	24.58	26.48	27.03	27.64	19.48	25.05	24.78	23.27	23.27	16.91	23.73	17.05	-	47	141	136	136	136	138	138	134	140	139	139	139	143
Seq66	Diplocolenus subg. Verdanus (1507-2019)	19.73	18.43	18.53	18.29	19.11	17.97	16.01	15.75	14.46	14.46	16.62	14.60	16.76	9.57	-	95	92	92	92	94	94	94	96	96	99	97	100
Seq29	Colladonus clitellarius (109-2018)	20.86	19.92	19.96	19.51	18.90	21.00	18.82	19.00	17.07	17.07	18.62	16.84	19.03	28.66	19.31	-	85	85	85	85	85	87	93	93	97	96	100
Seq10	Agallia sp. (72-2018)	19.73	18.64	18.74	18.50	20.12	21.65	19.74	19.91	17.72	17.72	20.34	17.85	20.45	27.70	18.74	17.28	-	0	0	2	2	2	79	79	82	80	88
Seq27	Agallia sp. (99-2018)	19.73	18.64	18.74	18.50	20.12	21.65	19.74	19.91	17.72	17.72	20.34	17.85	20.45	27.70	18.74	17.28	0.00	-	0	2	2	2	79	79	82	80	88
Seq59	Agallia sp. (1394-2019)	19.73	18.64	18.74	18.50	20.12	21.65	19.74	19.91	17.72	17.72	20.34	17.85	20.45	27.70	18.74	17.28	0.00	0.00	-	2	2	2	79	79	82	80	88
Seq19	Agallia sp. (86-2018)	19.73	18.64	18.90	18.46	20.08	21.65	20.09	20.26	18.05	18.05	20.34	17.44	20.45	27.99	19.07	17.24	0.41	0.41	0.41	-	0	4	79	79	83	82	88
Seq26	Agallia sp. (98-2018)	19.73	18.64	18.90	18.46	20.08	21.65	20.09	20.26	18.05	18.05	20.34	17.44	20.45	27.99	19.07	17.24	0.41	0.41	0.41	0.00	-	4	79	79	83	82	88
Seq14	Agallia sp. (78-2018)	19.73	18.64	19.14	18.90	20.53	21.65	19.78	19.96	17.76	17.76	20.34	18.26	20.45	27.40	19.14	17.68	0.40	0.41	0.41	0.80	0.80	-	81	81	84	80	90
Seq17	Endria inimica (1740-2019)	21.54	19.92	19.96	19.31	19.11	21.00	19.69	19.00	17.28	17.28	18.91	17.04	19.32	28.46	19.51	18.90	16.06	16.06	16.06	16.02	16.02	16.46	-	0	4	3	89
Seq68	Endria inimica (1586-2019)	21.54	19.92	19.96	19.35	19.14	21.00	19.74	19.04	17.31	17.31	18.91	17.07	19.32	28.31	19.55	18.94	16.09	16.09	16.09	16.06	16.06	16.50	0.00	-	4	3	89
Seq58	Endria inimica (1382-2019)	21.54	19.92	19.14	20.16	19.96	21.00	20.39	19.69	17.92	17.92	18.91	17.89	19.32	28.31	20.16	19.76	16.70	16.70	16.70	16.87	16.87	17.11	0.80	0.80	-	6	93
Seq45	Endria inimica (916-2019)	21.77	20.13	20.37	19.92	19.72	20.82	19.34	18.64	17.14	17.14	19.20	17.65	19.60	28.37	19.76	19.51	16.29	16.29	16.29	16.63	16.63	16.33	0.61	0.61	1.22	-	92
Seq56	Elymana inornata (1358-2019)	22.90	21.82	21.79	21.14	20.73	22.94	19.91	20.31	18.50	18.50	20.63	18.26	20.74	29.07	20.33	20.33	17.89	17.89	17.89	17.85	17.85	18.29	18.09	18.13	18.94	18.70	-
Seq23	Paraphlepsius sp. (90-2018)	22.00	20.76	20.77	19.72	22.36	20.35	18.82	19.00	16.67	16.67	19.48	16.43	19.32	28.86	20.33	19.72	19.11	19.11	19.11	19.07	19.07	19.51	18.70	18.74	19.55	19.31	17.48
Seq28	Paraphlepsius sp. (105-2018)	22.00	20.76	20.37	20.17	22.87	20.35	18.83	19.02	16.63	16.63	19.48	16.63	19.32	27.23	20.58	20.17	19.33	19.33	19.33	19.33	19.33	19.33	19.13	19.13	19.13	19.33	17.88
Seq4	Empoasca fabae (39-2018)	24.34	23.50	24.19	23.43	23.20	30.17	25.17	25.64	25.17	25.17	28.47	25.69	28.52	34.97	26.51	25.29	23.26	23.26	23.26	23.61	23.61	23.31	24.13	24.19	24.88	23.78	24.59
Seq9	Empoasca fabae (71-2018)	23.58	22.88	23.01	22.36	22.36	28.14	25.93	26.10	23.47	23.47	25.79	23.94	25.85	31.63	23.83	23.17	22.56	22.56	22.61	22.92	22.92	22.61	23.17	23.22	23.83	23.06	23.78
Seq7	Empoasca fabae (56-2018)	23.36	22.46	22.40	21.75	21.75	28.35	26.10	26.26	23.63	23.63	26.07	23.73	26.14	31.36	23.01	22.97	22.40	22.40	22.40	22.72	22.72	22.81	22.97	23.01	23.63	23.22	23.37
Seq39	Empoasca fabae (196-2018)	23.36	22.46	22.40	21.75	21.75	28.35	26.10	26.26	23.63	23.63	26.07	23.73	26.14	31.57	23.22	22.97	22.40	22.40	22.40	22.72	22.72	22.81	22.97	23.01	23.63	23.22	23.58
Seq43	Empoasca fabae (338-2018)	24.60	24.52	24.20	24.20	24.20	29.53	27.42	27.59	26.11	26.11	27.64	26.11	27.68	29.72	25.90	25.48	25.05	25.05	25.05	25.05	25.05	25.05	25.48	25.48	25.48	25.69	24.84
Seq16	Empoasca fabae (81-2018)	24.72	28.60	30.69	30.83	30.63	29.59	34.51	34.65	32.38	32.38	27.14	32.79	27.20	28.27	32.11	31.64	32.25	32.25	32.11	32.39	32.39	31.98	31.85	31.71	31.71	31.77	33.47
Seq42	Empoasca fabae (325-2018)	24.89	25.05	24.73	24.73	25.60	29.50	29.34	29.51	27.55	27.55	27.51	27.55	27.56	28.51	26.46	27.11	26.90	26.90	26.90	26.90	26.90	26.90	26.03	26.03	26.03	26.03	28.42
Seq36	Empoasca fabae (185-2018)	26.53	27.43	27.51	27.51	26.86	29.69	30.02	30.19	28.82	28.82	27.22	28.82	27.56	30.65	29.04	27.95	29.26	29.26	29.26	29.26	29.26	29.26	28.38	28.38	28.38	28.38	30.13
Seq32	Empoasca fabae (127-2018)	24.94	23.52	23.42	22.56	21.54	27.71	26.10	26.04	23.22	23.22	26.07	23.33	26.42	31.57	22.81	23.17	22.20	22.20	22.20	22.52	22.52	22.61	24.39	24.44	25.05	24.64	21.95
Seq34	Empoasca fabae (173-2018)	24.94	23.52	23.83	22.97	21.95	27.71	25.71	25.66	23.27	23.27	26.07	23.73	26.42	31.43	23.22	23.58	22.61	22.61	22.61	22.92	22.92	22.65	24.80	24.85	25.46	24.69	22.36
Seq13	Empoasca fabae (76-2018)	26.65	26.65	26.65	26.65	24.11	27.41	29.30	29.22	26.90	26.90	25.89	26.90	26.25	26.90	26.90	27.16	26.40	26.40	26.40	26.40	26.40	26.40	26.90	26.90	26.90	26.90	28.17
Seq60	Graphocephala hieroglyphica (1424-2019)	24.49	23.09	23.22	22.56	23.17	26.84	24.40	24.56	22.45	22.45	24.93	22.92	25.28	32.65	24.24	21.95	20.57	20.57	20.57	20.89	20.89	20.61	20.93	20.98	21.59	20.82	21.95
Seq15	Erythroneura sp. (79-2018)	22.90	21.61	21.59	20.73	22.56	25.54	23.63	23.80	21.14	21.14	23.50	20.89	23.86	31.10	21.75	20.73	19.72	19.72	19.72	19.68	19.68	20.12	21.54	21.59	22.40	22.15	19.51
Seq61	Forcipata loca (1435-2019)	20.86	19.92	19.96	18.90	20.73	24.89	23.41	23.36	21.14	21.14	23.50	20.89	23.58	31.50	22.76	20.93	17.68	17.68	17.68	17.65	17.65	18.09	21.75	21.79	22.61	22.36	21.34
Seq25	Graphocephala sp. (97-2018)	22.62	21.61	21.34	21.13	23.64	23.33	22.57	22.30	20.08	20.08	19.77	20.08	20.17	26.78	20.92	20.08	19.25	19.25	19.25	19.25	19.25	19.25	21.97	21.97	21.97	22.18	21.34
Seq31	Draeculacephala sp. (113-2018)	19.95	18.86	18.39	18.80	20.25	23.81	22.94	22.89	20.87	20.87	21.49	20.66	21.87	27.48	20.25	18.60	16.32	16.32	16.32	16.32	16.32	16.74	20.25	20.25	20.25	20.66	21.28
Seq64	Draeculacephala sp. (1475-2019)	19.95	18.64	18.74	18.50	19.72	23.81	22.37	22.32	20.37	20.37	21.49	20.49	21.87	28.31	19.76	18.09	16.29	16.29	16.29	16.63	16.63	16.70	19.92	19.96	20.57	20.16	21.34
Seq46	Draeculacephala sp. (921-2019)	20.18	19.07	19.35	18.70	20.12	24.03	22.64	22.59	20.61	20.61	21.49	21.10	21.87	28.57	20.37	18.90	16.50	16.50	16.50	16.84	16.84	16.53	20.53	20.57	21.18	20.41	21.54
Seq69	Doratura stylata (1588-2019)	21.09	20.13	20.57	20.53	21.95	21.43	21.44	21.62	19.11	19.11	20.06	18.86	20.17	27.03	19.51	20.53	19.47	19.47	19.51	19.47	19.47	19.88	19.92	19.76	20.37	20.53	20.53
Seq72	Doratura stylata (1707-2019)	21.09	20.13	20.16	19.72	21.14	21.43	20.57	20.74	18.29	18.29	20.06	18.05	20.17	27.03	18.70	19.72	18.70	18.70	18.70	18.66	18.66	19.11	19.11	19.14	19.96	19.72	19.72
Seq57	Jikradia olitoria (1378-2019)	24.26	23.09	23.01	22.56	22.56	24.46	22.32	22.27	19.92	19.92	22.35	19.68	22.16	28.66	19.92	18.90	18.09	18.09	18.09	18.05	18.05	18.50	21.14	21.18	22.00	21.75	20.33
Seq40	Cuerna sp. (238-2019)	25.00	23.27	23.70	22.78	24.51	28.07	24.34	24.07	23.91	23.91	26.73	24.03	26.79	32.61	23.91	24.08	22.83	22.83	22.83	23.16	23.16	23.26	23.43	23.48	24.13	23.48	24.51
Seq20	Empoasca fabae (1730-2019)	26.12	24.49	25.11	24.45	25.55	28.24	24.34	24.12	23.67	23.67	28.21	24.18	27.94	35.40	26.71	24.01	21.41	21.41	21.41	21.76	21.76	21.46	25.77	25.77	26.43	25.44	24.45
Seq35	Paraphlepsius sp. (179-2018)	28.90	27.37	27.66	26.79	29.45	30.28	22.78	23.38	26.43	26.43	32.08	26.53	31.81	36.68	28.28	30.27	27.87	27.87	27.87	28.16	28.16	28.28	29.45	29.51	30.12	29.71	26.79
Seq3	Cuerna sp. (43849)	26.53	28.51	28.14	28.35	29.00	27.33	30.21	29.91	27.92	27.92	26.65	27.92	26.70	26.94	25.97	27.27	25.76	25.76	25.76	25.76	25.76	25.76	26.84	26.84	26.84	26.68	27.27
Seq49	Cuerna sp. (964-2019)	29.80	27.87	27.95	27.23	27.02	29.37	25.38	25.33	25.27	25.27	29.75	25.00	29.78	33.12	23.97	25.05	23.97	23.97	23.97	23.91	23.91	24.40	23.97	24.02	24.89	24.40	23.75
Seq37	Idiocerus sp. (190-2018)	25.40	26.48	26.13	26.35	28.51	30.30	30.14	30.07	28.29	28.29	29.80	28.29	29.83	30.32	28.29	29.16	27.00	27.00	27.00	27.00	27.00	27.00	27.86	27.86	27.86	27.86	28.29
Seq38	Idiocerus sp. (194-2018)	27.44	29.05	28.67	28.88	31.29	32.17	32.94	32.86	31.29	31.29	30.95	31.29	30.97	31.37	30.42	31.29	28.67	28.67	28.67	28.67	28.67	28.67	30.85	30.85	30.85	30.85	30.42
Seq5	Empoasca fabae (50-2018)	33.26	33.41	32.95	32.95	34.55	35.93	38.81	38.71	35.24	35.24	30.40	35.24	31.27	33.64	33.41	35.24	36.16	36.16	36.16	36.16	36.16	36.16	34.32	34.32	34.32	34.55	34.32
Seq41	Empoasca fabae (322-2018)	33.33	34.07	33.62	33.62	34.71	38.61	39.20	39.11	35.79	35.79	30.37	35.79	31.25	35.50	33.62	35.79	36.23	36.23	36.23	36.23	36.23	36.23	34.49	34.49	34.49	34.71	33.84
Seq21	Empoasca fabae (88-2018)	29.50	29.98	29.89	29.68	29.89	33.12	33.88	33.33	31.61	31.61	31.05	31.61	31.36	32.98	30.97	33.12	30.54	30.54	30.54	30.54	30.54	30.54	28.60	28.60	28.60	28.60	29.03
Seq51	Latalus sp. (1424-2019)	28.34	27.12	26.48	27.03	27.85	32.25	30.85	30.79	28.05	28.05	32.66	27.99	32.67	34.55	26.02	30.49	28.46	28.46	28.46	28.60	28.60	28.86	30.28	30.35	29.94	30.69	28.25
Seq6	Aphrodes bicinctus (52-2018)	31.75	30.72	30.35	29.47	28.66	32.47	30.85	30.79	27.85	27.85	30.09	27.59	29.83	37.80	28.86	25.81	24.14	24.14	23.98	23.94	23.94	24.54	24.80	24.85	25.66	25.41	29.07
Seq12	Aphrodes bicinctus (120-2018)	32.65	32.80	32.35	32.35	32.81	32.81	32.68	32.60	30.77	30.77	31.81	30.77	31.53	33.41	32.35	30.09	27.15	27.15	27.15	27.15	27.15	27.15	27.60	27.60	27.60	27.83	32.35
Seq24	Aphrodes bicinctus (93-2018)	32.43	31.42	31.03	31.03	31.03	33.55	32.13	32.05	29.35	29.35	31.23	29.35	30.97	36.90	30.61	28.30	25.79	25.79	25.79	25.79	25.79	25.79	26.62	26.62	26.62	26.83	30.82
Seq47	Aphrodes bicinctus (933-2019)	32.20	31.14	30.96	30.28	30.28	33.33	31.21	31.14	28.37	28.37	30.95	28.80	30.68	38.37	29.74	27.64	25.05	25.05	25.05	25.35	25.35	25.10	26.02	26.07	26.68	25.92	30.08
Seq48	Aphrodes bicinctus (934-2019)	35.37	34.11	34.01	33.54	32.72	37.01	35.38	35.31	32.24	32.24	35.82	32.66	35.51	41.63	33.40	31.10	28.46	28.46	28.51	28.80	28.80	28.51	29.27	29.12	29.74	29.18	33.13
Seq11	Aphrodes bicinctus (73-2018)	34.08	34.08	34.08	33.80	32.96	32.96	32.68	32.96	32.40	32.40	32.66	32.40	32.33	32.96	32.96	30.73	27.93	27.93	27.93	27.93	27.93	27.93	28.77	28.77	28.77	28.77	32.68
Seq65	Aphrodes bicinctus (1506-2019)	38.91	39.46	38.94	38.94	38.27	40.27	40.05	40.19	37.83	37.83	40.11	37.83	39.77	41.19	39.60	37.39	33.19	33.19	33.19	33.19	33.19	33.19	33.85	33.85	33.85	34.07	38.27
Seq22	Empoasca fabae (89-2018)	33.25	32.36	32.10	32.46	33.55	36.83	32.82	33.62	33.99	33.99	35.44	33.91	35.74	40.52	33.55	33.99	30.50	30.50	30.50	30.65	30.65	30.94	32.03	32.10	31.88	32.24	31.15
Seq8	Paraphlepsius sp. (63-2018)	33.93	38.03	39.19	38.91	40.32	36.27	39.35	39.26	37.70	37.70	32.57	37.63	32.29	38.45	40.12	38.71	35.47	35.34	35.08	35.01	35.01	35.47	38.31	38.18	38.38	38.71	37.70
Seq44	Paraphlepsius sp. (416-2018)	33.63	33.26	33.26	33.03	34.16	33.94	33.17	33.33	33.26	33.26	34.38	33.26	34.37	34.62	34.39	33.48	33.03	33.03	33.03	33.03	33.03	33.03	33.26	33.26	33.26	33.26	33.03
Seq18	Paraphlepsius sp. (85-2018)	31.69	32.17	32.12	32.33	33.62	34.48	33.18	33.10	32.76	32.76	32.29	32.76	32.58	36.03	34.26	33.83	31.48	31.48	31.48	31.48	31.48	31.48	31.69	31.69	31.69	31.69	34.26

Table S3. Continued

Seq23	Seq28	Seq4	Seq9	Seq7	Seq39	Seq43	Seq16	Seq42	Seq36	Seq32	Seq34	Seq13	Seq60	Seq15	Seq61	Seq25	Seq31	Seq64	Seq46	Seq69	Seq72	Seq57	Seq40	Seq20	Seq35	Seq3	Seq49	Seq37	Seq38	Seq5	Seq41
97	97	92	104	103	103	109	109	110	117	110	110	105	108	101	92	100	88	88	89	93	93	107	102	105	126	117	121	112	121	146	147
98	98	98	108	106	106	114	135	114	124	111	111	105	109	102	94	102	89	88	90	95	95	109	104	108	130	130	124	121	131	144	155
102	98	104	113	110	110	114	151	114	126	115	117	105	114	106	98	102	89	92	95	101	99	113	109	114	135	130	128	121	131	144	155
97	97	101	110	107	107	114	152	114	126	111	113	105	111	102	93	101	91	91	92	101	97	111	105	111	131	131	125	122	132	144	155
110	110	100	110	107	107	114	151	118	123	106	108	95	114	111	102	113	98	97	99	108	104	111	113	116	144	134	124	132	143	151	160
94	94	121	130	131	131	137	137	136	136	128	128	108	124	118	115	108	110	110	111	99	99	113	121	120	139	126	126	140	147	157	178
86	84	108	118	119	119	119	157	125	127	119	117	109	111	108	107	100	103	102	103	98	94	102	111	110	105	129	116	129	139	156	167
87	85	110	119	120	120	120	158	126	128	119	117	109	112	109	107	99	103	102	103	99	95	102	110	109	108	128	116	129	139	156	167
82	80	108	115	116	116	123	159	127	132	114	114	106	110	104	104	96	101	100	101	94	90	98	110	107	129	129	116	131	143	154	165
82	80	108	115	116	116	123	159	127	132	114	114	106	110	104	104	96	101	100	101	94	90	98	110	107	129	129	116	131	143	154	165
68	68	82	90	91	91	97	95	96	95	91	91	87	87	82	82	69	75	75	75	70	70	78	85	88	111	93	94	104	108	107	106
81	80	111	118	117	117	123	162	127	132	115	117	106	113	103	103	96	100	101	104	93	89	97	111	110	130	129	115	131	143	154	165
68	68	83	91	92	92	98	96	97	97	93	93	89	89	84	83	71	77	77	77	71	71	78	86	88	111	94	95	105	109	111	110
142	131	150	155	154	155	140	132	132	141	155	154	106	160	153	155	128	133	139	140	133	133	141	150	160	179	125	152	141	144	147	164
100	99	114	117	113	114	122	158	122	133	112	114	106	119	107	112	100	98	97	100	96	92	98	110	121	138	120	110	131	139	146	155
97	97	109	114	113	113	120	156	125	128	114	116	107	108	102	103	96	90	89	93	101	97	93	111	109	148	126	115	135	143	154	165
94	93	100	111	110	110	118	159	124	134	109	111	104	101	97	87	92	79	80	81	96	92	89	105	97	136	119	110	125	131	158	167
94	93	100	111	110	110	118	159	124	134	109	111	104	101	97	87	92	79	80	81	96	92	89	105	97	136	119	110	125	131	158	167
94	93	100	111	110	110	118	158	124	134	109	111	104	101	97	87	92	79	80	81	96	92	89	105	97	136	119	110	125	131	158	167
94	93	102	113	112	112	118	160	124	134	111	113	104	103	97	87	92	79	82	83	96	92	89	107	99	138	119	110	125	131	158	167
94	93	102	113	112	112	118	160	124	134	111	113	104	103	97	87	92	79	82	83	96	92	89	107	99	138	119	110	125	131	158	167
96	93	100	111	112	112	118	157	124	134	111	111	104	101	99	89	92	81	82	81	98	94	91	107	97	138	119	112	125	131	158	167
92	92	104	114	113	113	120	157	120	130	120	122	106	103	106	107	105	98	98	101	98	94	104	108	117	144	124	110	129	141	150	159
92	92	104	114	113	113	120	156	120	130	120	122	106	103	106	107	105	98	98	101	97	94	104	108	117	144	124	110	129	141	150	159
96	92	107	117	116	116	120	156	120	130	123	125	106	106	110	111	105	98	101	104	100	98	108	111	120	147	124	114	129	141	150	159
95	93	102	113	114	114	121	156	120	130	121	121	106	102	109	110	106	100	99	100	101	97	107	108	115	145	123	112	129	141	151	160
86	86	106	117	115	116	117	165	131	138	108	110	111	108	96	105	102	103	105	106	101	97	100	113	111	131	126	109	131	139	150	156
-	0	105	109	108	108	115	152	117	129	103	105	104	109	100	93	105	97	97	98	102	98	93	106	104	118	117	105	123	128	142	153
0.00	-	103	107	107	107	115	141	117	129	102	103	104	107	100	93	105	97	96	96	98	98	93	105	102	117	117	105	123	128	142	153
24.36	24.52	-	4	6	5	4	48	18	19	69	67	55	82	95	86	83	103	104	101	115	111	107	94	89	107	115	105	100	107	66	83
22.15	22.25	0.93	-	4	3	9	52	21	30	70	70	61	92	102	91	90	107	108	105	125	121	110	107	94	141	126	116	114	123	66	83
21.95	22.25	1.40	0.80	-	1	10	52	21	30	68	70	60	94	100	90	90	106	107	106	123	119	109	103	95	141	126	114	116	125	66	81
21.95	22.25	1.16	0.61	0.20	-	9	51	20	29	69	71	60	93	101	90	90	106	107	106	124	120	109	104	95	140	127	115	115	124	65	82
24.42	24.42	0.98	1.91	2.12	1.91	-	35	26	34	77	77	66	100	108	97	98	114	115	113	127	127	116	105	95	143	133	116	120	129	71	87
30.83	29.25	11.19	10.57	10.57	10.37	7.42	-	29	51	113	112	60	136	145	139	124	145	151	150	166	165	153	143	135	182	136	157	193	130	69	92
25.38	25.38	4.50	4.56	4.56	4.34	5.62	6.24	-	32	78	78	62	103	112	105	105	117	116	116	128	128	118	108	103	151	135	121	121	127	67	88
28.17	28.17	4.79	6.55	6.55	6.33	7.39	9.46	6.93	-	88	88	59	111	118	113	112	125	125	124	139	139	130	113	108	156	134	124	214	131	72	94
20.93	21.21	16.05	14.26	13.85	14.05	16.35	22.97	16.92	19.21	-	2	63	82	92	86	97	99	98	99	127	123	111	104	95	129	126	114	113	122	101	109
21.34	21.41	15.62	14.29	14.26	14.46	16.35	22.81	16.92	19.21	0.41	-	63	82	94	88	97	101	100	99	129	125	113	104	93	129	126	116	113	122	101	109
26.40	26.40	15.90	15.48	15.23	15.23	16.67	15.23	15.74	14.97	15.99	15.99	-	86	91	83	77	89	89	89	108	108	98	100	89	134	110	113	97	99	101	98
22.15	22.25	19.11	18.78	19.14	18.94	21.23	27.70	22.34	24.24	16.70	16.73	21.83	-	94	83	76	89	87	87	113	109	118	97	106	135	116	107	115	124	131	145
20.33	20.79	22.04	20.73	20.33	20.53	22.93	29.41	24.30	25.76	18.70	19.11	23.10	19.11	-	87	88	96	96	97	99	95	108	98	99	128	122	108	106	111	135	145
18.90	19.33	19.95	18.50	18.29	18.29	20.59	28.19	22.78	24.67	17.48	17.89	21.07	16.87	17.68	-	80	85	87	86	113	109	104	107	90	128	123	111	112	118	137	152
21.97	21.97	19.90	18.83	18.83	18.83	20.76	25.89	22.73	24.40	20.29	20.29	19.54	15.90	18.41	-	-	77	77	76	102	102	102	92	106	141	110	101	117	123	132	144
20.04	20.17	24.35	22.11	21.90	21.90	24.20	29.90	25.38	27.29	20.45	20.87	22.59	18.39	19.83	17.56	16.11	-	2	2	97	97	96	88	97	138	123	115	116	128	143	155
19.72	19.96	24.19	22.00	21.79	21.79	24.42	30.69	25.16	27.29	19.96	20.37	22.59	17.72	19.51	17.68	16.11	0.41	-	4	102	98	95	88	98	138	124	115	116	128	143	154
19.92	19.96	23.54	21.43	21.59	21.59	23.99	30.55	25.16	27.07	20.16	20.20	22.59	17.76	19.72	17.48	15.90	0.41	0.80	-	104	100	99	88	95	138	124	118	117	129	142	154
20.73	20.37	26.68	25.35	25.00	25.20	26.96	33.60	27.77	30.35	25.81	26.22	27.41	22.97	20.12	22.97	21.34	20.04	20.73	21.14	-	4	106	109	113	146	129	125	122	127	152	166
19.92	20.37	25.75	24.59	24.19	24.39	26.96	33.47	27.77	30.35	25.00	25.41	27.41	22.15	19.31	22.15	21.34	20.04	19.92	20.33	0.80	-	102	105	110	142	129	121	122	127	152	166
18.90	19.33	24.83	22.36	22.15	22.15	24.63	31.03	25.60	28.38	22.56	22.97	24.87	23.98	21.95	21.14	21.34	19.83	19.31	20.12	21.54	20.73	-	116	111	156	128	116	123	131	150	161
22.99	23.33	21.86	23.26	22.39	22.61	23.97	31.09	25.12	26.46	22.61	22.61	26.60	21.09	21.26	23.21	20.58	19.43	19.13	19.13	23.64	22.78	25.16	-	88	98	111	100	91	102	146	155
22.91	22.97	20.79	20.80	20.97	20.97	21.99	29.87	24.29	25.65	20.97	20.58	24.05	23.45	21.81	19.82	24.04	21.70	21.63	21.02	24.89	24.23	24.45	19.43	-	101	122	112	92	100	127	135
24.13	24.48	24.60	28.89	28.89	28.69	30.56	37.30	32.97	34.36	26.43	26.43	33.58	27.66	26.18	26.18	29.68	28.69	28.28	28.28	29.86	29.04	31.90	21.08	22.05	-	147	114	127	132	178	188
25.32	25.32	28.68	27.27	27.27	27.49	28.66	29.18	29.09	29.07	27.27	27.27	27.92	25.11	26.41	26.62	23.76	26.62	26.84	26.84	27.92	27.92	27.71	25.75	28.71	32.03	-	27	109	116	154	170
22.88	23.44	24.36	25.27	24.84	25.05	26.61	34.20	28.27	29.18	24.84	25.27	30.21	23.31	23.53	24.18	22.70	25.50	25.05	25.71	27.23	26.36	25.27	21.79	24.67	24.57	6.29	-	107	114	155	164
26.57	26.57	24.88	24.62	25.05	24.84	25.81	33.68	26.08	36.27	24.41	24.41	24.62	24.84	22.89	24.19	25.22	25.05	25.05	25.27	26.35	26.35	26.57	21.06	21.60	27.61	23.70	24.88	-	28	144	153
28.01	28.01	27.02	26.91	27.35	27.13	28.10	28.26	27.61	28.54	26.70	26.70	25.13	27.13	24.29	25.82	26.86	28.01	28.01	28.23	27.79	27.79	28.67	23.94	23.81	29.07	25.27	26.89	6.10	-	151	168
32.49	32.49	17.55	15.10	15.10	14.87	16.17	15.75	15.33	16.48	23.11	23.11	25.44	29.98	30.89	31.35	30.14	32.72	32.72	32.49	34.78	34.78	34.32	35.96	31.75	41.01	35.24	38.37	32.95	34.55	-	14
33.19	33.19	20.75	18.00	17.57	17.79	18.79	19.87	19.05	20.48	23.64	23.64	24.87	31.45	31.45	32.97	31.17	33.62	33.41	33.41	36.01	36.01	34.92	36.05	31.84	41.05	37.20	38.32	33.48	36.68	3.22	-
30.32	30.32	22.64	21.94	21.72	21.72	22.91	21.70	22.01	23.13	19.35	19.35	19.54	26.45	26.88	27.31	27.96	27.74	27.53	27.53	31.40	31.40	30.11	28.01	27.93	35.29	31.20	30.70	30.64	32.33	27.89	28.97
27.03	26.82	32.48	30.28	29.88	29.67	31.85	37.93	33.19	35.59	28.46	28.86	33.50	30.49	29.47	29.27	27.41	27.27	27.64	28.05	29.47	28.86	28.46	30.15	34.36	36.40	33.55	32.68	31.53	33.70	40.05	41.21
27.64	28.27	29.23	27.18	26.83	26.83	29.51	36.03	31.67	32.97	27.24	27.64	28.17	28.46	28.25	24.19	25.52	26.45	26.22	26.63	28.40	27.44	23.98	30.59	28.85	36.20	32.90	30.94	33.26	34.79	39.59	39.91
30.54	30.54	30.97	29.64	29.86	29.86	30.63	31.53	31.53	32.73	30.77	30.77	29.95	31.90	31.45	27.60	28.67	29.41	29.41	29.64	30.54	30.54	27.15	31.87	31.11	38.95	32.96	33.74	32.51	33.86	40.96	40.41
29.35	29.35	30.29	28.30	28.30	28.30	30.36	34.73	32.54	33.84	29.35	29.35	29.95	30.19	29.77	25.58	26.78	27.88	27.88	28.09	28.93	28.93	25.79	31.39	30.23	37.13	33.98	32.88	33.48	35.01	40.73	41.00
28.66	28.90	29.37	27.35	27.49	27.49	30.15	36.25	32.32	33.62	28.51	28.57	29.70	29.18	29.07	25.00	26.57	27.69	27.29	27.35	29.07	28.25	25.20	30.65	29.42	36.27	33.77	32.03	33.26	34.79	40.50	40.78
32.52	32.43	33.80	31.16	31.36	31.36	33.76	39.84	36.01	37.34	31.77	31.84	34.26	32.04	32.11	29.67	30.54	30.79	30.75	30.82	32.45	32.11	29.67	34.35	31.86	39.14	36.80	35.29	35.64	37.20	44.39	44.47
31.28	31.28	30.37	30.17	30.45	30.45	30.45	30.45	30.73	31.01	31.28	31.28	29.97	32.40	31.84	28.77	28.49	30.17	30.17	30.17	31.56	31.56	28.49	31.28	30.73	35.26	33.52	32.96	33.80	33.80	42.66	42.18
36.50	36.50	38.62	38.50	38.72	38.72	39.65	41.23	41.01	42.20	37.83	37.83	39.34	38.94	38.72	35.62	35.76	35.84	35.84	36.06	37.61	37.61	34.29	40.14	40.00	42.98	40.44	40.81	40.66	42.29	49.54	49.45
26.80	26.79	29.00	30.07	30.50	30.28	30.28	37.47	32.01	33.41	26.58	26.58	30.21	31.15	28.32	27.89	31.69	29.71	29.85	30.28	31.81	31.15	29.85	29.85	27.97	31.68	34.50	31.59	23.95	25.47	35.89	36.21
31.05	29.90	42.17	41.45	41.53	41.53	40.21	41.42	39.48	39.96	41.73	41.73	35.95	41.33	38.91	38.51	36.93	38.52	39.52	39.31	40.44	40.32	38.10	39.44	42.89	43.00	37.45	40.48	39.52	39.83	44.90	45.55
23.76	23.76	34.65	34.39	34.62	34.62	35.81	36.12	34.09	35.52	34.84	34.84	34.60	34.62	32.81	32.81	32.51	33.94	33.94	33.71	34.84	34.84	33.26	31.14	35.06	36.45	34.16	34.72	34.39	34.62	43.51	42.99
30.41	30.41	32.35	32.33	32.12	32.12	33.05	32.62	32.26	32.47	32.55	32.55	28.86	34.48	31.26	32.12	30.56	31.05	30.84	31.26	32.76	32.76	31.69	28.05	32.87	39.52	32.62	32.56	31.12	33.62	39.82	41.59

Table S3. Continued

Seq21	Seq51	Seq6	Seq12	Seq24	Seq47	Seq48	Seq11	Seq65	Seq22	Seq8	Seq44	Seq18
131	125	140	144	143	142	156	122	172	135	151	149	141
137	128	145	143	148	147	161	122	176	144	181	145	148
139	130	149	143	148	152	167	122	176	147	194	147	150
138	133	145	143	148	149	165	121	176	149	193	146	151
139	137	141	145	148	149	161	118	173	154	200	151	157
154	149	150	145	155	154	171	118	182	158	169	150	161
145	141	141	133	142	142	161	117	167	150	181	135	143
143	141	141	133	142	142	161	118	168	154	181	136	143
147	138	137	136	140	139	158	116	171	156	187	147	153
147	138	137	136	140	139	158	116	171	156	187	147	153
109	114	105	111	109	108	125	97	140	112	114	120	113
147	138	136	136	140	142	161	116	171	156	187	147	153
111	115	105	111	109	108	125	97	140	114	114	121	115
154	170	186	148	176	188	204	118	187	186	183	153	169
144	128	142	143	146	146	164	118	179	154	199	152	160
154	150	127	133	135	136	153	110	169	156	192	148	158
142	140	119	120	123	123	140	100	150	140	177	146	147
142	140	119	120	123	123	140	100	150	140	176	146	147
142	140	118	120	123	123	140	100	150	140	174	146	147
142	141	118	120	123	125	142	100	150	141	174	146	147
142	141	118	120	123	125	142	100	150	141	174	146	147
142	142	121	120	123	123	140	100	150	142	177	146	147
133	149	122	122	127	128	144	103	153	147	190	147	148
133	149	122	122	127	128	143	103	153	147	189	147	148
133	147	126	122	127	131	146	103	153	146	190	147	148
133	151	125	123	128	127	143	103	154	148	192	147	148
135	139	143	143	147	148	163	117	173	143	187	146	160
141	133	136	135	140	141	160	112	165	123	154	105	142
141	129	136	135	140	139	156	112	165	120	145	105	142
91	140	126	118	126	126	145	106	151	125	183	132	131
102	149	134	131	135	134	153	108	174	138	206	152	151
101	147	132	132	135	135	154	109	175	140	206	153	150
101	146	132	132	135	135	154	109	175	139	206	153	150
107	150	139	136	143	142	159	109	180	132	191	159	155
102	187	178	140	166	178	196	109	188	172	198	160	153
103	153	146	140	150	149	166	110	187	137	184	151	151
108	163	151	145	155	154	171	111	192	142	185	157	151
90	140	134	136	140	140	156	112	171	122	207	154	152
90	142	136	136	140	140	156	112	171	122	207	154	152
77	132	111	118	118	117	135	107	155	113	142	137	114
123	150	140	141	144	143	157	116	176	143	205	153	161
125	145	139	139	142	143	158	114	175	130	193	145	146
127	144	119	122	122	123	146	103	161	128	191	145	150
130	131	122	127	128	127	146	102	162	141	178	144	143
129	132	128	130	133	134	149	108	162	134	188	150	145
128	136	129	130	133	134	151	108	162	137	196	150	144
128	138	131	131	134	134	151	108	163	139	195	149	146
146	145	140	135	138	143	160	113	170	146	201	154	153
146	142	135	135	138	139	158	113	170	143	200	154	153
140	140	118	120	123	124	146	102	155	137	189	147	148
121	139	141	131	140	141	158	112	169	137	183	128	122
119	156	131	126	133	133	144	110	166	127	196	142	141
162	178	177	171	176	177	191	128	193	147	212	160	183
146	155	152	146	157	156	170	120	184	148	173	151	152
132	150	142	138	146	147	162	118	171	145	187	142	141
144	146	154	144	155	154	165	121	185	103	183	152	145
150	154	159	150	160	159	170	121	192	108	184	153	156
123	175	173	179	178	177	194	154	217	145	198	191	176
135	190	184	179	189	188	205	151	224	155	210	190	193
-	172	157	155	161	160	176	125	191	148	205	179	169
36.99	-	156	153	154	159	175	133	184	150	231	175	158
33.76	31.71	-	21	18	19	45	14	82	164	215	177	165
34.60	34.62	4.84	-	8	7	45	13	71	147	170	176	156
34.62	32.29	3.84	1.84	-	1	42	15	73	157	205	180	165
34.41	32.32	3.93	1.61	0.21	-	43	14	73	162	216	179	164
37.85	35.57	9.28	10.37	8.96	8.92	-	41	94	183	231	194	182
34.92	37.15	3.98	3.69	4.26	3.98	11.65	-	60	124	129	133	119
41.52	40.71	18.22	16.06	16.22	16.22	20.89	16.76	-	184	192	198	191
34.42	32.68	35.73	35.94	35.36	35.29	39.87	34.64	43.91	-	213	158	145
43.71	46.57	43.26	38.03	42.62	43.55	46.48	36.03	42.01	46.10	-	125	174
40.04	39.59	40.05	39.82	40.72	40.50	43.89	37.05	44.70	38.63	28.03	-	161
36.03	33.83	35.33	34.82	35.33	35.12	38.97	33.24	41.61	33.49	37.34	36.02	-
